# Advancing Adult HPV Vaccination—Turning Evidence into Action

**DOI:** 10.3390/vaccines14050375

**Published:** 2026-04-23

**Authors:** Meera Gosalia, Michael Moore, Bettina Borisch, Marta Lomazzi, the members of the Global HPV Adult Vaccination Engagement Forum

**Affiliations:** 1 World Federation of Public Health Associations (WFPHA), Institute of Global Health, University of Geneva, Campus Biotech—G6, Chemin des Mines 9, 1202 Geneva, Switzerland; zchamsg@ucl.ac.uk (M.G.); mimomph@gmail.com (M.M.); bettina.borisch@wfpha.org (B.B.); 2 University College London Medical School, Gower St, London WC1E 6BT, UK; 3 Discipline of Public Helath, Health Research Institute, University of Canberra, Bruce, ACT 2617, Australia; 4 Institute of Global Health, University of Geneva, Campus Biotech—G6, Chemin des Mines 9, 1202 Geneva, Switzerland

**Keywords:** human papillomavirus (HPV), HPV vaccination, cervical cancer elimination, universal vaccination, health equity, cancer prevention, public health policy, global health

## Abstract

Human Papillomavirus (HPV) is one of the most prevalent infections worldwide and a leading cause of cervical cancer, as well as anal, oropharyngeal, penile, vulval, and vaginal cancers. Despite the availability of safe and effective vaccines, coverage beyond female adolescent programmes remains often insufficient, leaving many adolescents and adults unprotected. The World Federation of Public Health Associations (WFPHA) convened a year-long global expert engagement forum to develop evidence-informed policy recommendations to advance HPV elimination. Building on this work, the resulting Call-to-Action urges countries to expand access to boys and adults. Adopting a life-course approach, integrated with screening, equitable access policies, and sustainable financing, can significantly increase coverage and reduce the burden of HPV-related cancers. This article outlines the main outcomes of the Call-to-Action and highlights key priorities for policy and decision makers committed to accelerating HPV elimination.

## 1. Introduction

Human Papillomavirus (HPV) is a common global sexually transmitted infection [1] with an estimated 80% of men and women likely to acquire the virus at some point in their life [2]. It is an important cause of several cancers, many of which may remain latent for years [3]. High-risk HPV types are responsible for nearly 100% of cervical cancers and about 90% of anal cancers and also contribute to cancers of the oropharynx, penis, vagina, and vulva [4]. Individuals remain at risk of acquiring new infections with the same or different HPV genotypes during their life. HPV transmission dynamics differs between the two sexes with variation depending on population, context and HPV type [5]. Most infections naturally resolve with viral clearance occurring more rapidly in men [6]. As a result, men may unknowingly transmit the virus while remaining asymptomatic, contributing to ongoing circulation of HPV. Despite the availability of highly established and effective vaccines, global vaccine coverage remains insufficient, with full-dose coverage estimated at around 20% for females and 6% for males [7], leaving millions at continued risk and allowing preventable cancers to persist.

HPV vaccines provide protection against HPV strains not yet encountered by an individual [8], making vaccination especially valuable. It is also the only preventive option for certain HPV-associated cancers for which no screening programmes are available, offering significant potential to reduce the burden of HPV-related cancers in both sexes [9]. The absence of standardized screening for men also leaves many unaware of its risks [10]. Importantly, natural immunity following prior HPV infection does not provide full protection against reinfection with other HPV genotypes [3], meaning that vaccination remains beneficial even for individuals who have previously been infected.

Currently, there are six licenced prophylactic HPV vaccines, five of which have been WHO pre-qualified and the sixth (Cervavac^®^) is still seeking WHO pre-qualification [11] but is nationally licenced in India [12]. The licenced HPV vaccines have shown to be effective against HPV, all of which differ primarily in the number of HPV strains they protect against [13]. The three bivalent vaccines, (Cervarix™, manufactured by GlaxoSmithKline Biologicals, Rixensart, Belgium; Cecolin^®^, manufactured by Xiamen Innovax Co., Ltd., Xiamen City, Fujian Province, China; Walrinvax^®^ manufactured by Walvax Biotechnology Co., Ltd., Yuxi City, Yunnan Province, China), protect against high risk HPV types 16 and 18, which are responsible for causing nearly 70% of cervical cancers, but remains ineffective against genital warts [14]. Gardasil^®^, manufactured by Merck Vaccines as a quadrivalent HPV vaccine, protects against an additional two strains, HPV types 6 and 11, which cause approximately 90% of genital warts. The nonvalent vaccine, Gardasil 9^®^, also manufactured by Merck & Co., Inc. (Rahway, NJ, USA) and licenced most recently in 2014, offers the broadest protection and covers the additional HPV types 31, 33, 45, 53 and 58 [15].

Many national immunization programmes primarily target adolescents, increasingly including both girls and boys; however, vaccination strategies for adults remain limited in most countries [16]. Many adults were not vaccinated during adolescence or were ineligible under earlier guidelines. In response, several countries have implemented catch-up programmes for unvaccinated adults, expanding eligibility to 26 years or 45 years in some cases and no upper age limit in others [17]. Expanding vaccination also supports broader public health goals, including the World Health Organization target of eliminating cervical cancer as a public health problem by 2030 through the 90–70–90 strategy [18]: 90% of girls fully vaccinated against HPV by age 15, 70% of women screened with a high-performance test by ages 35 and 45, and 90% of women identified with cervical disease receiving appropriate treatment. Achieving these targets would not only reduce the burden of HPV-related diseases but also lower long-term healthcare costs associated with their treatment and management [19].

Disparities in access, awareness, and vaccine uptake persist across countries and population groups, particularly in LMICs where the burden of HPV disease is highest [20]. Recent global estimates (2023) highlight considerable regional variation, with full vaccination coverage highest in the WHO European region (60.9%) with lower uptake in the WHO South-East Asia region (28.9%) [21]. However, some low-income countries, including Rwanda and Uganda, have achieved comparatively high coverage rates compared with high income countries. Thought to be largely attributable to targeted pilot programmes and demonstration projects focused on women [22], these examples highlight the potential impact and success of focused investment and structured implementation, even in resource-constrained settings.

Multiple factors contribute to these persistent inequalities in coverage, including constraints with healthcare infrastructure, vaccine hesitancy, knowledge deficiency, sociocultural barriers, and competing health priorities [23,24]. Additionally, delivery strategies such as the integration of the HPV vaccine in national supportive measures and school-based policies has varying degrees of effectiveness in countries where existing funding is already limited for school health programmes. In contrast, countries such as Sweden demonstrate the potential of sustained investment and high vaccination coverage, with a national ambition of HPV-elimination by 2027 [25]. These patterns emphasize the need for context-specific strategies that address structural barriers while leveraging successful implementation models.

In conjunction with global health priorities, expanding HPV vaccination contributes towards wider healthcare targets, including cervical cancer elimination targets, life-course immunization strategies and universal health coverage. Despite strong evidence of its effectiveness [26], the HPV vaccination still remains underutilized in many settings, particularly beyond adolescent programmes. Addressing this gap requires deliberate, coordinated, cross-sector action to extend vaccination opportunities whilst maintaining high coverage among adolescents, who remain the primary prevention priority. Efforts must be supported by sustainable financing, strengthened delivery infrastructure, and equitable access for medically and socially vulnerable populations. Embedding vaccination into routine healthcare interactions can help normalize uptake and reduce missed opportunities. Therefore, a coordinated policy approach is essential to enable these changes. Strategies should incorporate culturally and linguistically responsive engagement to build trust, alongside robust monitoring mechanisms and registries to track uptake, coverage, and outcomes.

A consensus on the definition, scope, and policy positioning of adult HPV vaccination is required to support evidence-based recommendations and strengthen the basis for national policy development. In the absence of such alignment, variations in policy continue to limit implementation and access, slowing progress in reducing the burden of HPV-related diseases beyond adolescence.

This article outlines and presents key outcomes of the WFPHA Call-to-Action with aims to translate emerging evidence into actionable policy recommendations. It highlights key policy priorities and practical strategies for implementation to accelerate progress toward HPV elimination.

## 2. Materials and Methods

To address this public health issue, the World Federation of Public Health Associations (WFPHA) has established a high-level Engagement Forum bringing together 44 global experts in HPV from diverse fields, including screening, immunization, treatment, policy, and public health. Over the course of one year, this group met regularly (twice face to face, five times online in addition to regular exchanges via email) to develop a comprehensive Call-to-Action: Advancing HPV Vaccination for Adults—From Evidence to Action (hereafter Call-to-Action), which has since been presented at key regional and international meetings [27].

## 3. Priority Policy Areas for Stakeholder Engagement

A series of policy areas for stakeholder discussion has been identified by the experts to support dialogue on the development of policies that advance universal HPV vaccination, meaning vaccination programmes that include both sexes and extend beyond adolescents to adults. The areas presented in this article are not ranked in order of priority. All are considered important, and countries should determine their priorities based on their specific economic, demographic, political, and health system contexts. However, adopting a life-course approach to vaccination (vaccination spanning all stages of life) together with a universal approach (ensuring equitable access to all eligible populations: girls, boys, adults) should be considered a foundational starting point. For the purpose of this Call-to-Action, adults refer to individuals aged 19 years and older, with policy relevant subgroups including young adults (19–26 years), mid adults (27–45 years) and adults older than 45 years.

First, adopting inclusive terminology is essential to strengthening HPV vaccination efforts and promoting equitable access. Framing vaccination as a universal intervention that includes males and females, adolescents and adults, can help shift perceptions away from risk-based or gender-restricted approaches and reduce associated stigma. Such reframing can also support broader public acceptance and normalize vaccination across the life course.

Second, particular attention should be paid to vulnerable and underserved populations. Adult men remain an underserved population under many current HPV vaccination strategies [10], despite facing a continued lifetime risk of infection. As many infections in men remain asymptomatic [28], male carriers can contribute to ongoing transmission, potentially undermining existing vaccination efforts. Progress toward reducing HPV-related morbidity therefore requires addressing these transmission dynamics during the life course [29].

Third, policy development should be informed by lessons from countries that are approaching HPV elimination. For example, Sweden’s “fast-track” approach [30] illustrates how comprehensive vaccination strategies can accelerate progress toward elimination. Effective adult vaccination programmes must incorporate delivery models that enable convenient access and sustained engagement across diverse populations. Where there may not be a formal national vaccination programme, opportunistic and targeted outreach vaccine models can be integrated into routine healthcare interactions with pharmacies and sexual health services, thereby reducing missed opportunities at different stages of life [31]. As vaccine supply and infrastructure improves, a phased and context appropriate expansion can complement rather than compromise efforts to vaccinate adolescent girls. Robust monitoring systems are also essential. Tracking adult vaccination coverage, uptake patterns, and health outcomes will be critical to evaluating programme effectiveness and guiding policy refinement.

Fourth, inclusion of stakeholders from low- and middle-income countries (LMICs)—where the burden of HPV-associated disease is greatest—is also essential. Policy development and implementation must reflect the realities of these settings. Strengthening local data systems should therefore be a sustained priority, supported through partnerships with non-profit organizations and technical agencies to establish registries and adapt monitoring approaches in contexts with limited infrastructure. Without deliberate investment in capacity and leadership from LMICs, global HPV prevention efforts risk reinforcing existing inequities rather than reducing them.

Fifth, reliance on risk stratification to guide HPV vaccination in adults is unlikely to deliver a meaningful public health impact. Lessons can be drawn from the transition of hepatitis B vaccination from risk-based to universal coverage [32,33], which significantly expanded protection. For example, in Taiwan, the introduction of hepatitis B vaccination at birth in 1986 has prevented millions of chronic infections and significantly reduced the incidence of hepatocellular carcinoma in children [34], while in the United States it has led to a 99% decline in pediatric hepatitis B virus infections since 1991 [35]. Given the ubiquitous nature of HPV and the unpredictable patterns of exposure throughout an individual’s life [3] attempts to vaccinate only those identified as “high risk” are inherently limited.

This challenge is further compounded by current public funding schemes, which often rely on fixed age-based criteria and may exclude adult populations who could still benefit from vaccination. Evidence suggests that HPV vaccination up to the age of 45, can be economically justified in the long term, especially when integrated with screening and supportive policies [36]. Cost-effectiveness is also likely to improve alongside the number needed to vaccinate, when vaccination is targeted towards higher-risk mid-adult groups, including individuals with multiple sexual partners, those who have recently separated, and women who are under-screened [37]. A modelling study conducted in Italy supports that expanding vaccination to older age groups (up to 45 years) prevents significant mortality and morbidity with negligible additional costs [36]. This is supported, with varying optimal age ranges, by previous older studies conducted across Belgium, Japan, China, the Netherlands, the UK, and the USA [38,39,40,41]. Additionally, HPV-positive women have a substantially higher long-term absolute risk of recurrent CIN2+ compared to HPV-negative women. Supporting this, a retrospective study found that 90% of post-surgical vulvar, vaginal, or anal lesions were associated with vaccine-covered HPV types. Therefore peri-treatment prophylactic 9vHPV vaccination has also been shown to be a cost-effective intervention to prevent subsequent HPV-related diseases and manage lower genital tract lesions among adult patients undergoing surgical treated for cervical precancer (CIN2+) [29,42].

A shift toward pragmatic, access-driven models is therefore needed to normalize vaccination opportunities within routine care. This may include delivery through pharmacies, workplaces, and community-based venues to reduce logistical barriers. In parallel, sustained education and advocacy by healthcare professionals will remain essential to encourage vaccine uptake and ensure consistent engagement with adult populations.

Finally, vaccination strategies should not exclude individuals with prior HPV exposure. Adults who have previously been infected with one or more HPV types can still benefit substantially from immunization against the same or other circulating genotypes, as vaccination provides more consistent and durable protection than natural infection [16]. Expanding vaccination opportunities to include adults with prior exposure is therefore a logical step toward reducing HPV prevalence and preventing future infections.

## 4. Discussion

Recognizing the global burden of HPV-related diseases and cancers, successful policy models and strategies from countries progressing toward elimination should be adapted for broader global application. At the same time, it is important to emphasize that in settings where financial or logistical constraints limit programme expansion, priority should always be given to girls and adolescents, who bear the highest burden of HPV-related cervical cancer. In resource-constrained settings, the pace and scope of expansion will need to be carefully balanced against competing health priorities and available financing. However, given the increasing burden of anal, oropharyngeal and other HPV-related cancers, a comprehensive vaccination strategy that includes boys and adults should also be prioritized when possible. Such an approach would strengthen programme resilience in the event of disruptions to vaccination services, such as those observed during the COVID-19 pandemic [30]. Universal vaccination strategies across genders, age groups, and socioeconomic contexts represent the most equitable and sustainable model for long-term disease control. In line with principles of health equity and global immunization commitments, a universal approach is ultimately the only strategy capable of delivering equitable, sustainable, and population-level reductions in HPV-related diseases.

The expansion of HPV vaccination should therefore be implemented through a life-course immunization strategy rather than as a siloed intervention. A life-course approach supports vaccination opportunities throughout an individual’s life, including for those previously unvaccinated and for individuals with prior HPV exposure, recognizing that vaccination provides protection beyond that conferred by natural infection. Combined with robust screening programmes, early diagnosis pathways, and equitable access to treatment, this integrated approach would maximize not only the return on investment but also population health outcomes and quality of life for those affected by HPV-related cancers. Indeed, while cost-effectiveness remains highest in adolescents, vaccination strategies extending to adults, including targeted higher-risk groups, and peri-treatment settings, are economically justified in the long term. In countries without an established HPV national immunization programme, pragmatic approaches should be considered such as opportunistic vaccination through primary care and other community-based services. Implementation pathways should therefore remain context-specific, reflecting differences in epidemiology, health system capacity, and resource availability. Targeted outreach may also be incorporated to further enhance uptake in settings with limited infrastructure.

Beyond its role as convener of the expert engagement process, WFPHA is actively supporting the dissemination and implementation of the Call-to-Action by building a broad network of well-recognized organizations endorsing the initiative, thereby strengthening its advocacy impact and credibility. In parallel, WFPHA is organizing a series of meetings and events to engage public health professionals, healthcare providers, and other stakeholders, fostering dialogue and facilitating the translation of recommendations into practice.

## 5. Conclusions

HPV remains a preventable cause of a substantial global burden of cancer, and this Call-to-Action urges governments, health leaders, global stakeholders, and national public health associations to adopt a phased expansion of HPV vaccination programmes to include adults. Moving beyond risk-based and age-restricted frameworks will help reduce transmission through herd immunity and close coverage gaps, particularly in low- and middle-income countries (LMICs). Achieving disease elimination will depend on sustained political commitment and financing, inclusive communication strategies, and robust monitoring mechanisms. Together, these actions provide a coordinated pathway toward equitable protection and accelerated elimination of cervical and other HPV-related cancers worldwide.

## Data Availability

No new data wes created or analyzed in this study. Data sharing is not applicable to this article.

## Abbreviations

The following abbreviations are used in this manuscript:

HPV	Human Papillomavirus
WFPHA	World Federation of Public Health Associations
WHO	World Health Organization
LMIC	Low and Middle-Income Countries
COVID-19	Coronavirus Disease 2019
